# Evaluation of Groundwater Quality for Human Consumption and Irrigation in Relation to Arsenic Concentration in Flow Systems in a Semi-Arid Mexican Region

**DOI:** 10.3390/ijerph18158045

**Published:** 2021-07-29

**Authors:** Jennifer Ortiz-Letechipia, Julián González-Trinidad, Hugo Enrique Júnez-Ferreira, Carlos Bautista-Capetillo, Sandra Dávila-Hernández

**Affiliations:** Doctorado en Ciencias de la Ingeniería, Campus UAZ Siglo XXI, Universidad Autónoma de Zacatecas, Carretera Zacatecas-Guadalajara Km. 6, Ejido La Escondida, Zacatecas 98160, Mexico; jenniol@uaz.edu.mx (J.O.-L.); baucap@uaz.edu.mx (C.B.-C.); sandra.davila@uaz.edu.mx (S.D.-H.)

**Keywords:** arsenic, flow systems, groundwater, water quality, drinking and irrigation

## Abstract

The supply of drinking water to the population is an important challenge facing humanity, since both surface and underground sources present a great variability of water storage with respect to space and time. This problem is further aggravated in arid and semi-arid areas where rainfall is low and torrential, which makes groundwater the main source of supply; therefore, it is necessary to carry out studies that allow evaluating the evolution of the quantity and quality of water. This study addresses the behavior of groundwater in a semi-arid region, considering the theory of flow systems to identify movement as well as water quality, es determined by a water quality index (WQI), calculated considering arsenic and fluorine. In addition, a quality irrigation classification is used, employing the norms in accordance with international standards and the Mexican Norm, which allows for a comparison. Local, regional, intermediate and mixed flow systems are identified, and the evolution of cations and anions in addition to temperature is examined. It is observed that the drinking water quality index classifies them as excellent in most of the monitored wells (<50), but with a negative evolution. Regarding irrigation, most of the water samples are classified without restriction for the establishment of any type II crop (C_2_S_1_) and with restrictions for horticultural crops. It is observed that arsenic had values between 0.49 and 61.40 (µg/L) in 2005, while in 2015 they were between 0.10 and 241.30 (µg/L). In addition, fluoride presented values between 0.00 and 2.6 (mg/L) in 2005, while in 2015 they were between 0.28 and 5.40 (mg/L). The correlations between arsenic and fluorine are noted as well as WQI and SAR. A finding in this research was to include arsenic and fluorine in the calculation of the WQI allowing a better interpretation of the quality of water for both human consumption and for agricultural use to based on this make the best decision to control any harmful effects for the population, in addition to identifying the appropriate purification treatment required to control pollutants. It is concluded that arsenic is an element of utmost importance when considering water quality, so it is necessary to examine its evolution and continue to monitor its levels constantly.

## 1. Introduction

Water is an essential and extremely important compound for life, as most organisms contain between seventy and ninety percent of it. The majority of fresh water is underground [1]. Groundwater tends to be sweet, and usually suitable for human consumption, however, sometimes, pollutants reach the aquifer due to natural factors (if the aquifers are too rich in dissolved salts or by natural erosion of certain rock formations) or human activities (such as septic tank construction or agriculture) [2].

The global demand for water is influenced by population growth, urbanization, food and energy security policies and macroeconomic processes, such as trade globalization and changing consumption patterns. Over the past century, water resource development has been driven by the population’s demand for food, and energy. The rising incomes and living standards of an expanding middle-class population have led to a sharp increase in water use, which could become unsustainable, especially where the supply is vulnerable or scarce [3].

The most vulnerable regions, such as arid and semi-arid areas, face the challenge of maintaining sufficient water sources to meet the demands of their main uses (agricultural, urban, livestock, industrial uses, among others). Groundwater continues to be an essential and reliable source of supply of water and because climate change has generated great spatial–temporal precipitation variability and has an effect on the storage volume in water reservoirs, this behavior is even more critical in arid regions [4,5].

Developing the understanding of the mechanisms that govern groundwater movement, considering the hydrological cycle behavior and its spatiotemporal variation will allow for groundwater exploitation policies to be developed, as by doing so, it will be possible to infer recharge volumes in aquifers and their rock–water interactions that define water quality and the extraction volumes for different uses [6,7].

There are many researchers who have studied the water recharge processes in different types of aquifers, mainly in arid and semi-arid areas. The variables that have been considered are isotopy of groundwater, geological behavior of the environment, transmission losses in natural channels, water–water interaction, vegetation, remote sensing, modeling approaches, land use, urban artificial recharge, among others [8,9,10,11,12]. Another approach that is used is the methodology called the flow system, which considers the fact that the recharged water in a specific area can have a route that can be used in a different region from that of the recharged water. In this research three flow systems can be considered: (1) the local flow system, in which the water infiltrates and quickly emerges to the soil surface (characterized by low temperatures (±10 °C)); (2) the intermediate flow system, which is located at ±80 m depth with a temperature of ±25 °C; and (3) the regional flow system, which presents high concentrations of chlorides and temperatures of ±40 °C [9,13,14]. 

For the development of all its activities (physiological, recreational, hygienic, economic and social) humans require a good quality of water, and the term “water quality” (WQ) reflects the physical and chemical water components determined when assessing the suitability of water. There are a number of factors that directly influence water quality, such as dissolved minerals, concentration of microscopic algae, pesticides and herbicides, heavy metals, and other contaminants [15]. One of the main natural elements present in groundwater is arsenic, which has become a global problem, as several world regions present significant levels of this element in drinking and domestic water [2]. World Health Organization (WHO) [16] standards regarding water quality objectives were established in relation to the individual components of drinking water that can cause health risks as a result of long-term exposure and where fluctuations in concentration are small [17,18,19,20].

Arsenic contamination (natural or anthropogenic) in drinking water has posed a serious risk to the health of billions of people around the world [20,21,22,23]. Throughout the world, several countries are affected: more than 24% in Africa (15 of 61 countries); 37% in America, (21 of 57 countries), 59% in Asia (33 of 56 countries), 67% in Europe (34 out of 51 countries), and 11% in Oceania (4 out of 35 countries) [24]. Among these, the most affected region is Asia, where more than 120 million people are exposed to arsenic, followed by America and Africa, where more than 48 and 24 million people are exposed to it, respectively [24,25,26]. Drinking contaminated water is responsible for 80% of all illnesses and deaths in developing countries [27,28].

Arsenic in drinking water can cause a number of adverse health effects, such as characteristic skin lesions that often appear relatively shortly after exposure (within 5 to 15 years of ingestion), and long-term exposure damages to various internal organs that can lead to bladder, lung and, skin cancers [29,30]. The WHO has stipulated an As concentration limit in drinking water of <10 µg/L [31]; however, there is still considerable uncertainty about the health risks due to exposure to low concentrations of As, and each country has different regulations with different permissible levels of arsenic in drinking water [32].

There is no information regarding arsenic ingestion via water consumption in the Mexican population for the year 2021; however, it has been recorded that approximately 2.0 million inhabitants ingest water with concentrations ranging from 0.030 to 0.590 mg/L in some regions of the country. The localities whose water supply sources are contaminated with this metalloid are located in the states of Chihuahua, Coahuila, Durango, Sonora, Nuevo León, Baja California Norte, Sinaloa, San Luis Potosí, Zacatecas, Aguascalientes, Guanajuato, Jalisco, Morelos, Hidalgo, and Guerrero [33,34,35], most of which are located in the north-central region of Mexico.

The aims of this research, which was carried out during two monitoring periods in the Calera aquifer in the state of Zacatecas, Mexico, were as follows: (1) to identify different flow systems and evaluate them for human and agricultural consumption; (2) to determine the water quality and its hydrogeochemical characteristics for human and agricultural consumption; and (3) to perform a statistical analysis with the data collected by applying the bivariate data analysis system (BiDASys). The results of this study are expected to contribute to the implementation of policies produced by decision makers based on scientific information regarding the quality of irrigation water used and for human consumption.

## 2. Materials and Methods

### 2.1. Study Area

**Figure 1:**
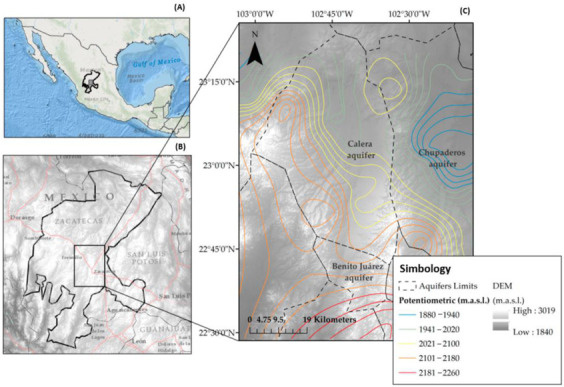
(**A**) Study area: the state of Zacatecas in Mexico. (**B**) Digital elevation model (DEM) with the territorial extension of the Calera. (**C**) Benito Juárez and Chupaderos administrative aquifers.

The study area is located in the volcanic terrain of the Sierra Madre Occidental in the Mexican highlands, which are located within the southern part of a graben structure region that gives rise to the endorheic basin of Calera. There is important surface runoff that is stored by hydraulic infrastructure that was built in the region. Zacatecas (maximum elevation of 2700 m.a.s.l.) and the Chilitos geological formation are delimited by a plateau area (2010 m.a.s.l.) in the south-central part and other plateau areas (2100 m.a.s.l.). Gypsisol soils predominate, and lithosol and regosol are characteristic of the semi-arid areas, with depths ranging between 10 and 200 cm. The predominant climate is considered arid with temperatures that vary between −9 °C in winter and 35 °C in summer with an average of 18 °C; the precipitation is spatially varied within a range of 100 to 600 mm, and the largest amount of rain occurs during the summer. The metropolitan area called Zacatecas-Guadalupe, with a population of 500,000 (±500) inhabitants, has an important agricultural area where approximately 25,000 hectares are irrigated with mainly garlic, chili, and onion crops. The necessary water for the different sectors (industrial, agricultural, livestock, urban) comes from the aquifers that are located in this area; therefore, we are interested in monitoring the water quality to determine if it meets the standards for these uses (Figure 1) [36].

The National Water Commission (CONAGUA) took several factors into consideration (geopolitical limits, areas with a high volume of wells and hydrological basins, among others) in order to define the 653 groundwater management units (called administrative aquifers (AAs)) throughout the country. Among these, 34 AAs were established in the state of Zacatecas [37]. This area is considered semi-arid, and the average annual rainfall (400–450 mm) is less than the maximum potential annual evaporation. These regions are characterized by a shortage of water, with a very irregular distribution of rainfall and some torrential events [13]. 

### 2.2. Geological Features

Four geological formations are located within the state of Zacatecas: the Sierra Madre Occidental, Sierra Madre Oriental, Mesa del Centro, and Eje Neovolcánico. In Zacatecas, there are igneous, sedimentary, and metamorphic rocks, whose formation ages correspond from the Triassic to the Recent. The oldest are low-grade metamorphic rocks (phyllites, shales, and schists). However, those with the greatest territorial distribution are the igneous rocks of the Tertiary (andesites, tuffs, rhyolites, and basalts) that outcrop in most of the Sierra Madre Occidental. The sedimentary rocks of the Mesozoic (Jurassic and Cretaceous) form folded structures (anticlines and synclines) that, in turn, have been dislocated by fractures and faults. Cenozoic igneous rocks appear with typical structures (volcanic devices and lava flows) and in the form of intrusive bodies that affect pre-existing rocks [38]. Geophysical studies and direct drilling show that the aquifer is unconfined and locally semi-confined by interbedded clay layers. The depth of the basement is estimated to be between 400 and 500 m. It is made up of a polymictic conglomerate, predominantly rhyolite and quartz fragments, with clay cementation and with re-deposit of clay tuffs [39,40].

### 2.3. Sample Collection and Concentration Determination

A campaign was carried out with 215 groundwater samples, each one from a different well around the aquifer of Calera in Zacatecas, Mexico. The evolution of groundwater quality was analyzed for a period of 10 years (2005−2015), for different wells. Temperature (T), electrical conductivity (EC), total dissolved solids (TDS), hydrogen potential (pH), and dissolved oxygen were measured in the field, using an isolation cell to prevent atmospheric interaction and achieve stability in the electrodes reading. An alkalinity test was performed, which provides information about the bicarbonate system that is subject to dissolution-precipitation processes, making it convenient to analyze the water in the place. For alkalinity proposes, a HACH^®^ (Hach Company, Loveland, Colorado, United States) device that includes cartridges containing H_2_SO_4_ at 0.16 normal concentration (N) was used; equilibrium point determination was carried out with phenolphthalein indicators and bromophenol blue indicators from which the concentrations of the CO_2_ and HCO_3_^−^ ions were obtained. All samples were collected in plastic bottles without air bubbles to stabilize it, and they were filtered (0.45 μm), ensuring the elimination of dissolved solids that could affect subsequent determinations of major ions and trace elements. The acidification process was also carried out (1% *v*/*v* HNO_3_^−^), and samples were transported and stored at a temperature of 4 °C [41].

Analytical determinations were carried out in the Environmental Engineering Laboratory of the Autonomous University of Zacatecas. Atomic absorption spectrophotometry (ICE AA 3300, Thermo Fisher Scientific, Waltham, MA, USA with generation of hydrides was also performed there. The determination of major ions Ca^2+^, Na^+^, K^+^, and Mg^2+^ was conducted using the same equipment as that used for As. Chloride was determined by titration using AgNO_3_ and K_2_CrO_4_ indicators. Other anions were determined by colorimetry, SO_4_^2−^ by precipitation of BaSO_4_, and N-NO_3_^−^ by the automated cadmium reduction method. Total alkalinity as HCO_3_^−^ was determined by titration using H_2_SO_4_, phenolphthalein, and bromophenol blue indicators. Calibrations for atomic absorption spectrophotometry and automated colorimetry were performed using an appropriate dilution standard; both laboratory and international reference materials were used for precision checks (4 sigma). Additional control included ion balance below ±7%. The precision of the physicochemical parameters was verified using the ionic equilibrium error (EBI), and the cations and anions are expressed as meq/L (Equation (1)) with a permissible limit of ±10%:

(1) $$\mathrm{EBI}=\frac{\sum \;anions-\sum \;cations}{\sum \;cations+\sum \;anions}*100$$

All determinations were made under the guidelines described in APHA-SMWW 2006: Standard Methods for the Examination of Wastewater [41] and applicable Mexican regulations.

### 2.4. Data Analysis

#### 2.4.1. Flow Systems Identification

The system flow theory used in the current study is described by Tóth [42], who considered groundwater flow distances and the geochemistry of water. Groundwater flow is controlled by various factors, such as geological and hydrogeological factors, and these are represented in terms of horizontal movement distance and depth. These hydraulic characteristics produces aquifer areas with a specific water quality called hydrochemical facies, and, therefore, it is feasible to establish a difference between them. For this research, the flow systems reported by Avila-Sandoval et al. [43] were used with some modifications.

#### 2.4.2. Drinking Water Quality 

Hydrogeochemical analysis (numerical and graphic analysis) was performed in the AquaChem 9.0 program designed by Waterloo Hydrogeologic (Waterloo company, Waterloo, ON, Canada). 

For the permissible limits of each parameter, the guidelines of the World Health Organization (WHO) [31], the U.S. Environmental Protection Agency (US EPA) [30], the Mexican Official Norm NOM-127-SSA1-1994 [44] and the Bureau of Indian Standards (BIS) [45] were considered (Table 1). 

The water quality index (WQI) was developed by Brown et al. and it assumes that the weighting of various water quality parameters is inversely proportional to the recommended standards for corresponding parameters [15,46]. The calculation procedure consisted on three stages. In the first stage, each of the ten parameters (pH, TDS, Cl^−^, SO_4_^2−^, HCO_3_^−^, F, Ca^2+^, Mg^2+^, As, Na^+^, and K^+^) is assigned a weight (*Wi*) based on its perceived effects on primary health. The maximum weight of five is assigned to parameters such as total dissolved solids, chloride, and sulfate due to their great importance in evaluating water quality (Table 2).

In the second stage, the relative weight (*Wi*) of each parameter is calculated using Equation (2):

(2) $$w_{i}=\frac{w_{i}}{\sum_{i=1}^{n}W_{i}}$$

where *w_i_* is the weight of each parameter, *n* is the number of parameters, and *W_i_* is the relative weight. The weight (*w_i_*), the calculated relative weight values (*W_i_*), and the standards were measured considering WHO parameters.

In the third stage, a quality rating scale (*qi*) is calculated for each parameter using Equation (3):

(3) $$SI=W_{i}\times q_{i}$$

A final summation is performed, and the calculated WQI values are generally classified into five categories: excellent (<50), good (50–100), fair (100–200), poor (200–300), and unacceptable (>300) for human consumption [13,18,47,48].

#### 2.4.3. Irrigation Water Quality

Agriculture in arid and semi-arid regions dependent on irrigation, but the hot and dry climate requires that the irrigation water does not contain soluble salts that could damage crops or that have an adverse effect on soil properties. Water of such quality is often not available in sufficient quantities to meet the crop water requirements. In this study, the water quality for agricultural use was obtained using the Richards classification [49] for irrigation water based on electrical conductivity (EC) and other important parameters described below.

The sodium adsorption radio (SAR) can indicate the alkalization ability of groundwater—the higher this value, the stronger the alkalization ability—and it was obtained with Equation (4) as follows:

(4) $$\mathrm{SAR}=\frac{{\mathrm{Na}}^{+}}{\frac{\sqrt{{\mathrm{Ca}}^{2+}+{\mathrm{Mg}}^{2+}}}{2}}$$

Sodium content is often expressed in terms of percent sodium or soluble sodium percentage (%Na); is significant in classifying irrigation water due to the decrease in soil permeability as a result of its reaction with soil [50]; and can obtained with Equation (5):

(5) $$\%\;\mathrm{Na}=\frac{\left({\mathrm{Na}}^{+}+K^{+}\right)}{{\mathrm{Ca}}^{+}+{\mathrm{Mg}}^{2+}+{\mathrm{Na}}^{+}+K^{+}}\times 100\%$$

The magnesium hazard (MH) suggested by Szaboles and Darab [51] was used to assess the suitability of agricultural irrigation, and it indicates that if Mg^2+^ content in irrigation water reaches a certain level, the MH may influence the soil, which would affect its structure and produce toxic effects [50,52]. It was obtained with Equation (6):

(6) $$H=\frac{{\mathrm{Mg}}^{2+}}{{\mathrm{Ca}}^{2+}+{\mathrm{Mg}}^{2+}}\times 100\%$$

The cumulative presence of salts in huge amounts in the soil zones may destroy the soil structure and reduce soil permeability. The permeability index (PI, Equation (7)) was also used as a criterion to estimate quality suitability for irrigation [50]. Table 3 shows the limits for each index previously described. Wilcox diagrams were produced in AquaChem 9.0.

(7) $$\mathrm{PI}=\frac{{\mathrm{Na}}^{+}+\sqrt{{\mathrm{HCO}}_{3}^{-}}}{{\mathrm{Ca}}^{2+}+{\mathrm{Mg}}^{2+}+{\mathrm{Na}}^{+}}\times 100\%$$

#### 2.4.4. Statistical Analysis

For the statistical analysis of arsenic and other parameters, the Bivariate Data Analysis System software (BiDASys, UNAM, CDMX, Mexico) was used to identify the linear regression models of least-squares weighted by uncertainty (OLR and UWLR). The uncertainty weighted least-squares linear regression (UWLR) model is a new weighted linear regression procedure based on estimates of total uncertainty. It is considered a good alternative, because the use of uncertainty has a probability connotation, a strict confidence level of 99%. For this calculation, the software uses the studentized residuals for both linear regression models (OLR and UWLR) using the following equation (Equation (8)):

(8) $$Sr_{j}=\left|\frac{r_{j}}{\sqrt{\frac{\sum r_{j}^{2}}{n-2}}\sqrt{\left(1-\frac{1}{n}\right)-\left(\frac{{\left(x_{j}-\bar{x}\right)}^{2}}{\sum {\left(x_{j}-\bar{x}\right)}^{2}}\right)}}\right|$$

where *Sr_j_* represents the studentized residuals; *r_j_* the residuals calculated by each linear regression; *n* the number of samples; and $x_{j}$ and $\bar{x}$ the individual value and the mean from sample *x*, respectively. Five recursive discordancy tests with the highest detection power and with the lowest skewness and masking effects were applied to detect possible discordant outliers in the studentized residuals from the bivariate samples. OLR and UWLR were used to estimate the correlation between arsenic and other parameters in the different flow systems determined [53].

## 3. Results and Discussion

This research was carried out during two monitoring periods in the Calera aquifer in the state of Zacatecas, Mexico, where it was identified different flow systems and evaluate them for human and agricultural consumption; determined the water quality and its hydrogeochemical characteristics for human and agricultural consumption; and performed a statistical analysis with the data collected by applying the bivariate data analysis system (BiDASys). 

### 3.1. Identification of Flow Systems

**Figure 2:**
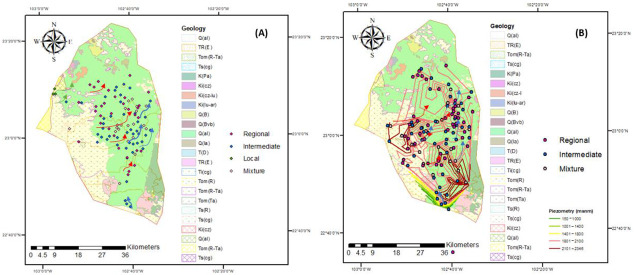
Corresponding wells in each flow system in the years (**A**) 2005 and (**B**) 2015.

The groundwater in the study area is the most important source of human, agricultural, and industrial water supply, which is why its evolution has previously been studied using the theory of flow systems. Avila et al. [43] identified regional, local, intermediate, and mixed flows through cluster analysis to group samples into groups that allow for the determination of the flow systems for the years 2005 and 2015, the results of which are shown in Figure 2. For 2005, 99 samples were analyzed from different wells—32 belonged to regional flow, 44 to intermediate flow, 3 to local flow, and 20 to mixed flow. (Table 4). On the other hand, for 2015 116 wells were evaluated as a result of cluster analysis—47 belonged to regional flow, 49 to intermediate flow, and 20 to mixed flow (Table 5). 

The method in this research was used with some modifications, such as consideration of the depth of the exploitations, for the evaluation of the suitability of groundwater quality for domestic use and irrigation in the period of 2005−2015. This type of study has not previously been carried out in this region, but it is necessary to evaluate the quality of water here in order to achieve sustainability, as, currently, is extracted on a large scale to meet the human consumption demands of approximately 500,000 inhabitants and for the irrigation of 25,000 hectares. [43]. One of the challenges of the scientific world is finding the origins of arsenic contamination, which can have different sources depending on the study region, requiring research in all of the planet to create a database that allows us to record the behavior of this element. Thereby helping to make necessary recommendations for the consumption of this type of water and preventing the deterioration of human health. In this research, the average arsenic content was higher in 2015 than in 2005 for the regional flow system (the monitoring of the 2016−2020 period presents very similar results; however, in this report they are not presented, because the data are currently in process of being analyzed), impacting the quality of water for human use. The origin of arsenic may be natural, due to water–rock interactions, as rocks such as pyrite, chalcopyrite, galean and marcasite contain it, and this type of geological material is present the study region. Other anthropogenic sources may also contribute to the contamination of the water with arsenic, through pesticides, herbicides, fertilizers or mining activities. The results obtained in this research indicate that the origin of arsenic depends on pyrite rock found in the region and is incorporated through water–rock interactions, that is, it occurs naturally. On this basis, it is necessary to apply remediation techniques, such as biosorbents or the design of wells for the extraction of water, avoiding harmful effects on the consumers of these waters.

The evolution of the cations in 2005 for the regional flow was Na^+^ > Ca^2+^ > K^+^ > Mg^2+^; for the remaining flow systems, the behavior is Ca^+2^ > Na^+^ > K^+^ > Mg^2+^. On the other hand, for the anions in all the flow systems, the evolution was presented as HCO_3_^−^ > SO_4_^2−^ > Cl^−^. In 2015, the evolution of the cations for the regional flow had the trend Na > Ca > Mg > K; It is observed that Mg^2+^ displaces K^+^, impacting on a deterioration of the water quality, for the remaining flow systems, there is Na^+^ > Ca^2+^ > K^+^ > Mg^2+^, that is, a very similar behavior. On the contrary, for anions in all flow systems, the present evolution is HCO_3_^−^ > Cl^−^ > SO_4_^2−^ having an effect on water quality. Most of the samples showed a similar behavior in terms of pH, Chebotarev [54] and others [1,42,55] have reported this cationic evolution.

Figure 2 shows the corresponding wells for each flow system in both years. It is shown that the wells are located in the Calera aquifer, which is one of the main aquifers that supply the metropolitan area of the state of Zacatecas for human consumption. Water is also extracted for the irrigation of crops that are consumed by the inhabitants. The flow system theory used in the present study has been described by Tóth [42], who considered groundwater flow distances and water geochemistry. However, from 2015, the depth of the extracted water was also incorporated, which varies from 10 to 300 meters.

### 3.2. Water Quality for Human Use

**Figure 3:**
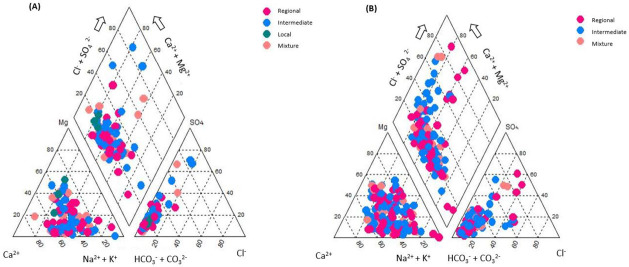
Piper diagram for the determination of the hydrogeochemical phases in each of the samples for the different flow systems: (**A**) 2005 and (**B**) 2015.

A Piper diagram is a graphical representation of some chemical components of water samples. Figure 3 shows the corresponding diagram of the different flow system in each year analyzed. It indicates that calcium, sodium, and/or magnesium bicarbonate are present. It can be seen in this diagram that for both years, the type in most of the samples has similar behavior [56]. This type of water is a result of the reaction between sodium chloride and silica that arise from the earth’s core in the presence of water, initially forming sodium silicate and then sodium bicarbonate of water [12]. These results agree with those of others studies [15,57,58].

**Figure 4:**
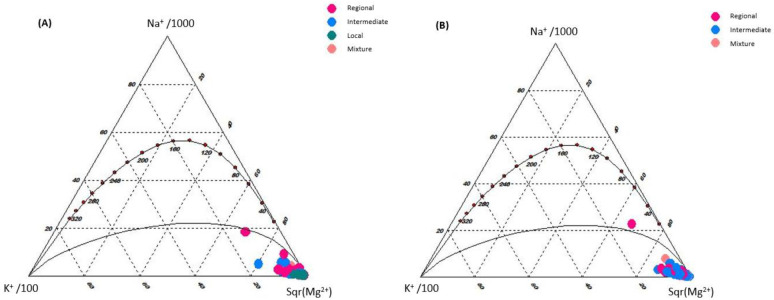
Giggenbach diagram of the different flow systems: (**A**) 2005 and (**B**) 2015.

Figure 4 shows the Giggenbach triangular diagram. This scheme reveals that almost all samples are in the immature water area, which is one of the main properties of cold groundwater. This type of water is characterized by not reaching equilibrium; that is, it does not have chemical equilibrium with respect to the rock of the aquifer, where dissolution dominates and it mixes with groundwater and ion exchange occurs. This result confirms that the chemical composition of groundwater is mainly controlled by the chemical dissolution of the rock [59], which is consistent with previously conducted studies [12,60].

Table 6 shows the average values of the parameters for each flow system observed in both years, and these are compared with different regulatory bodies around the world. The check mark indicates that these values are within the limits suggested by each regulation, while the cross indicates that these limits were exceeded and do not comply with the established standard. For the year 2005, most of the samples are within the permissible limit, with the exception of arsenic and nitrates that do not meet the standards of the World Health Organization. On the other hand, in 2015, a negative evolution was observed in these same parameters, thus not complying with the permissible limits of the EPA, and the samples of the mixed flow in arsenic do not comply with the Mexican standard. The scientific challenge of evaluating water quality has generated a series of studies in different regions of the world, such as India, USA, China, Switzerland, and Spain [22,61,62,63,64]; however, they do not consider flow systems that will allow for better visualization of quality of the water in aquifers, and can provide information for implement remediation methods that can prevent health problems, for the consumption of water with bad quality Therefore, such consideration represents innovation of the current investigation.

**Figure 5:**
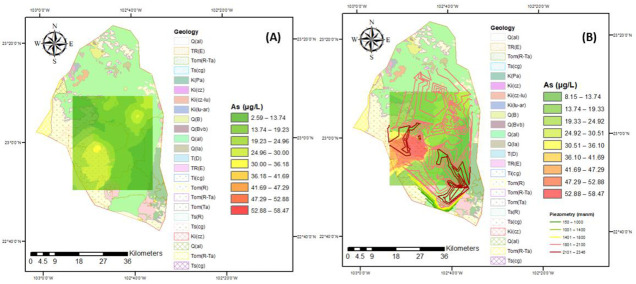
Corresponding arsenic concentration in the years (**A**) 2005 and (**B**) 2015.

**Figure 6:**
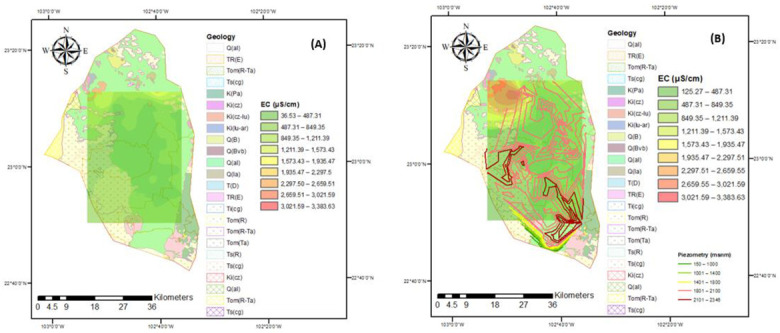
Corresponding EC in the years (**A**) 2005 and (**B**) 2015.

The evolution of arsenic concentrations can be observed in Figure 5, where problems with this element begin to appear since 2005, mainly in the southwest region of the Calera aquifer, for 2015 this trend continues but with significant increases in arsenic decreasing the quality of water for human consumption and irrigation. In 2015, the variable depth of water extraction was included, finding that the greater increase in the presence of arsenic, suggesting that the water extracted from this area should be restricted for human consumption as well as in the irrigation of agricultural crops to avoid Harmful effects on the population that drinks and consumes food, in addition to understanding the evolution of arsenic, it allows looking for treatment alternatives for the elimination of this element. On the other hand, electrical conductivity (Figure 6) has a similar behavior to arsenic since it increases its value from 2005 to 2015 in the north of the aquifer, the effect that this parameter may have on public health is due to the increase in concentrations of salts that damages the human system, for which a treatment is required for its control. On the other hand, some crops do not tolerate high concentrations of salts. 

**Figure 7:**
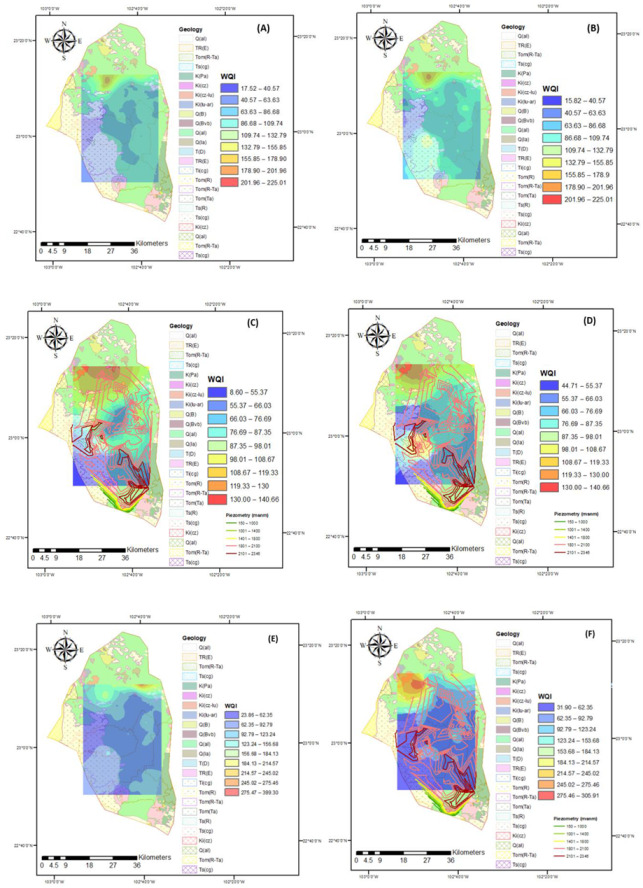
WQI calculated considering different parameters in the Calera aquifer sampling area: (**A**) WQI without considering arsenic in 2005; (**B**) WQI considering arsenic in 2005; (**C**) WQI without considering arsenic in 2015; (**D**) WQI considering arsenic in 2015; (**E**) WQI considering arsenic and fluor in 2005; (**F**) WQI considering arsenic and fluor in 2015.

The water quality index can be seen in Figure 7. Three indices were calculated, one without considering As, the other taking it into account, and the final one considering arsenic and fluor. For 2005, it can be observed how this index worsens when considering As in the calculation, and this can also be seen for the year 2015, which shows that it is an element of great importance for water quality and should be paid attention. These results indicate that according to the WQI, the groundwater is safe for drinking purposes; however, the quality of the water diminishes over time, with samples for 2015 belonging to the category of “unacceptable” in this year, which was not the case for 2005. Thus, it is recommended that this type of water is not used as drinking water in the region. The water quality index (WQI) is considered the most effective method of measuring water quality; however, while most studies do not consider arsenic as an important parameter, in this study, it can be observed that it is an element relevant to the quality of the water. Furthermore, a detailed analysis of water quality according to flow systems is considered to provide a better overview in order to describe the evolution of water quality in aquifers. Water quality assessment is important for pollution control and water resources management, and it is critical to identify the major contributors to spatial and temporal variations in water quality. 

The water quality indices in Figure 7 are based on the parameters that are considered high risk for human health [46]; however, several scientific works have suggested including other parameters to more accurately estimate the WQI, thereby avoiding adverse effects on the health of the population that consumes this water. In this investigation, the WQI was obtained by considering the parameters recommended in some investigations [15,46,47,48,64]. The second calculation of the WQI includes arsenic, which is considered a high risk element for human health if that contains a higher-than-average amount of it is consumed, according to the WHO [31]. The estimation of WQI with the arsenic and fluorine parameters allows us to generate a value with greater significance for decision makers regarding water use. In 2005, a change in the WQI without As and with As was observed, thus allowing for an increase in the level of classification of water quality. In 2015, a similar behavior occurred; however, an increase in the WQI was observed, that is, there was an evolution in the deterioration of water quality. This research shows that including As in the calculation of the WQI is of great importance since it allows a significant improvement in the classification of water quality, in addition to coinciding with other investigations that have been carried out [47,48,65,66]. It is therefore important to continue to monitor As as well as its origins and, based on this, recommend control or remediation techniques for As. On the other hand, the results reported in the research indicate that the WQI has a spatio-temporal behavior since some values change; however, according to this index, the groundwater in the study area is classified as safe to drink. Despite this being the case in most of the samples analyzed, some samples from 2015 belong to the category of “unacceptable”. The water quality index (WQI) is considered the most effective method for measuring water quality [46,52,67,68]. However, although most studies do not consider arsenic as an important parameter, in this study, it can be observed that it is a relevant element for water quality. Furthermore, a detailed analysis of water quality according to flow systems is considered to provide a better overview in order to describe the evolution of water quality in aquifers. The evaluation of water quality must be approached with spatial–temporal variability, thereby allowing for the control or remediation of heavy metals and the management of water resources.

### 3.3. Irrigation Water Quality

**Figure 8:**
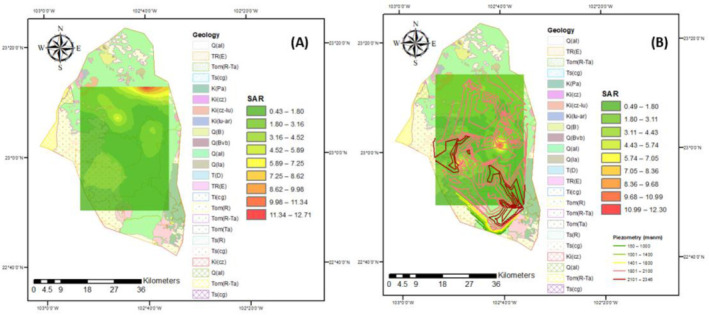
SAR for the years (**A**) 2005 and (**B**) 2015.

**Figure 9:**
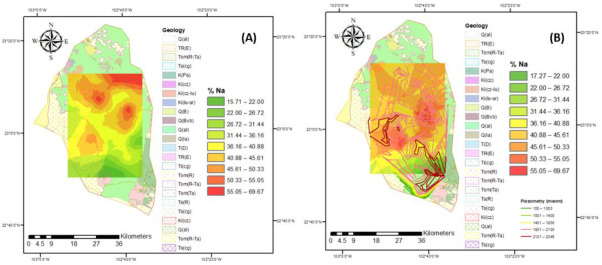
%Na present in the Calera aquifer around the sampling area for the years (**A**) 2005 and (**B**) 2015.

**Figure 10:**
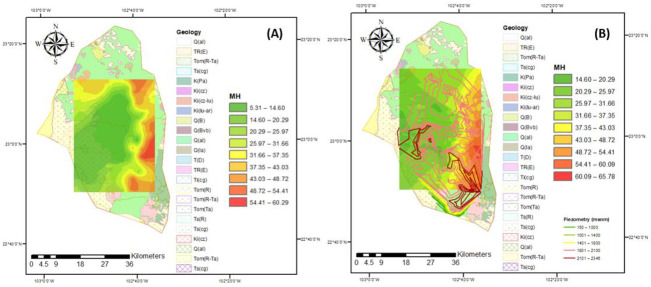
MH index for the years (**A**) 2005 and (**B**) 2015.

**Figure 11:**
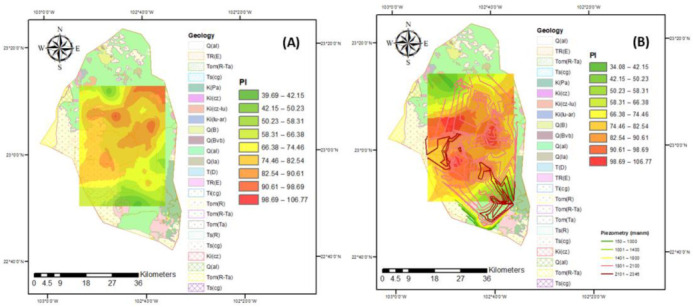
PI index for the years (**A**) 2005 and (**B**) 2015.

**Figure 12:**
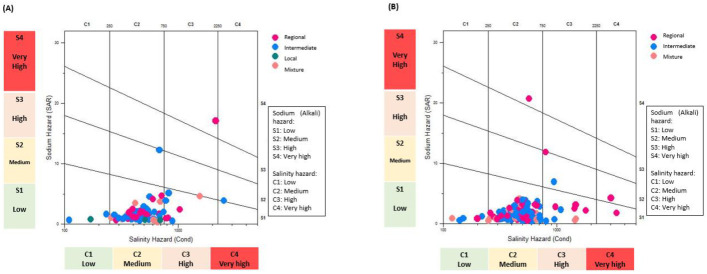
Classification of groundwater samples by means of the Wilcox diagram of the different flow systems: (**A**) 2005 and (**B**) 2015.

Groundwater in the study region is the main source of water to meet the irrigation needs of evapotranspiration for each crop in the area. Approximately 25,000 hectares of land growing predominantly vegetables and cereals are irrigated in two agricultural cycles (spring–summer and autumn–winter). It is estimated that 125 million m^3^ of water is annually extracted. To obtain the maximum yield, crops require comprehensive agricultural management, where water quality plays a fundamental role since it intervenes in all production processes. Groundwater is generally more mineralized with dissolved salts, which influence permeability, texture, structure, soil pH, the assimilation of nutrients, and crop growth. Poor-quality irrigation water directly influences crop yield, it is estimated that it decreases the yield of N by up to 35%, and, depending on the quality, some crops (those least tolerant to salinity) cannot be cultivated. The growth of vegetables also relies on this water, hence the importance of permanently monitoring and evaluation of the evolution of irrigation water quality. The FAO recommends some criteria for their classification and evaluation of their effect on soil and crops. The indices to classify the quality of groundwater for agricultural irrigation in the study region for the different flow systems are presented in Figure 8, Figure 9, Figure 10, Figure 11 and Figure 12.

Quality of groundwater can be affected by the use of fertilizers and pesticides. The pumping of water, the drilling of wells, and agricultural and mining activities cause intermediate and regional flows to mix, causing high concentrations of arsenic, which constitutes a risk to the population’s water supply. Groundwater is also influenced by natural aspects, such as salinity (Cl^−^, SO_4_^2−^, and Na^+^), redox conditions (Fe and Mn), age (F^−^ and B), and geology (As).

In the study area, the largest amount of water that is extracted is used for agriculture (approximately 78%). In this region, there is a 41-year-old agricultural operation where inorganic fertilizers of the nitrogen type have been applied. It is observed that these they have not caused contamination of the aquifer, as the values are below the Mexican norm. In this study, in order to evaluate the irrigation suitability of water quality, the EC index of water is used to present the hazard of salinity [69]. Most of the groundwater samples from this study area were categorized as good according to the EC for irrigation in the different flow systems in 2005 and 2015; however, in 2015 there was a stain with unsuitable values in the northwest region (Figure 6). 

Almost all of the samples for the different flow systems and years belonged to the “excellent” category according to the criteria of the SAR, with some exceptions presented in red zones where samples belonged to the “good” category (Figure 8). These results show the alkalization ability of the groundwater; in this case, high sodium ion content is not present, and, as such, it is very unlikely that the permeability of the soil is affected, thereby causing infiltration problems with the use of this water for irrigation. 

There has been an issue in regard to sodium percentage (%Na) since 2005, because most of the samples were classified as permissible and doubtful for agricultural use. This problem appears to worsen, increasing in the region with a high percentage of sodium in the aquifer, leaving in 2015 only a small area to the south with good values. This constitutes an important factor when determining groundwater quality for irrigational purpose, because excessive sodium content in groundwater could make the soil dense and impervious as a result of increasing the osmotic pressure and limiting the circulation of air and water to plants (Figure 9). 

Figure 10 presents the values for the MH index in the aquifer, and most of the values present desirable concentrations; however, for both years, a strip to the east of the aquifer shows values belonging to the category of undesirable for this parameter (Figure 10). These percentages indicate that there was an increase in magnesium content, with a greater number of wells affected in 2015. The magnesium hazardous (MH) ratio is an important parameter used to assess groundwater suitability for irrigation purposes, because high levels of Mg^2+^ in groundwater exchange with Na^+^ in soil. resulting in alkalization, which, consequently, decreases the crop productivity of plants.

Through calculation and analysis, it was found that PI values in 2005 and 2015 for the different flow systems of the groundwater samples were in the “moderately suitable for irrigation” category; however, these values present a negative evolution through the years (Figure 11). This implies that all of the groundwater sampling with the different flow systems were suitable for long-term agricultural irrigation purpose, displaying minimal influence on soil properties [70]. Moreover, the cumulative presence of salts in huge amounts will not destroy the soil structure and reduce soil permeability, thereby allowing the water to be suitable for agricultural uses.

The relationship between electrical conductivity (EC) and SAR was identified by utilizing a Wilcox diagram to classify groundwater quality for irrigation purposes, as shown in Figure 12. For both years (2005 and 2015), most of the samples were placed in the “C_2_-S_1_” category. 

The electrical conductivity of water (C_2_) indicates that some crops, such as vegetables, may present a decrease of 50% in their production, and some of them may not tolerate these concentrations of salt. As such, it is advisable in the aquifer regions to avoid cultivating these crops [18]. However, an evolution is shown in 2015, as some samples are classified as C_3_ and C_4_, S_3_, and S_4_, mainly in the aquifer region, suggesting that crops tolerant to salinity and sodicity are established in these irrigated areas, perhaps as a result of comprehensive soil management to avoid accumulation of salts.

The United States Salinity Laboratory (USSL) diagram has been used to study the quality of groundwater suitability for irrigation purposes [69]. As shown in Figure 12, the majority of the samples for both years fell into the C_2−_S_1_ (medium salinity with low sodium hazard) category, where using groundwater for irrigation would not produce sodium damage, and according to the Wilcox diagram, a large number of water samples were within the good-to-excellent category.

Not much attention has been paid to As exposure via food, especially rice and vegetables, which have been reported to contain high inorganic As concentrations in areas with elevated As in soil and irrigation water. Human exposure to As in contaminated regions may be very high due to the high As concentrations in groundwater (drinking and cooking water), and its high content in local agricultural produce is also likely. Plant uptake of As may be high on land irrigated with contaminated groundwater, thus illustrating the importance of establishing a good monitoring of the quality of water, not only for drinking purposes but also for irrigation in agriculture. The risk of arsenicosis is clearly the highest for disadvantaged persons, often women and children in poor families, due to inadequate food and nutrient intake [71,72].

### 3.4. Bivariate Data Analysis

**Figure 13:**
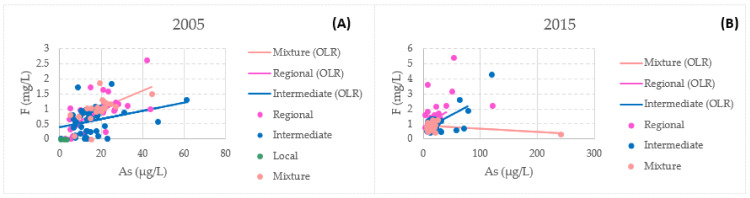
Uncertainty weighted least-squares linear regression (arsenic and fluor) for the different flow systems: (**A**) 2005 and (**B**) 2015.

**Figure 14:**
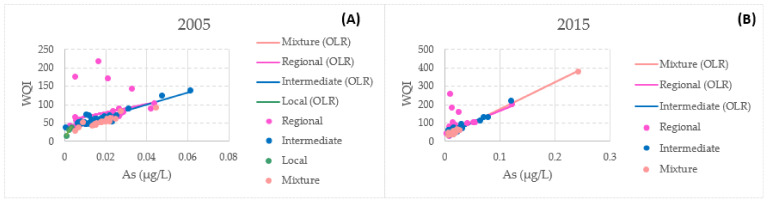
Uncertainty weighted least-squares linear regression (arsenic and WQI) for the different flow systems: (**A**) 2005 and (**B**) 2015.

**Figure 15:**
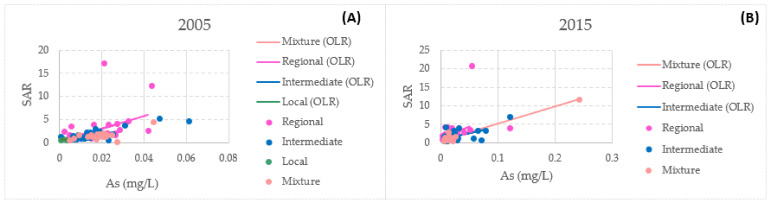
Uncertainty weighted least-squares linear regression (arsenic and SAR) for the different flow systems: (**A**) 2005 and (**B**) 2015.

The BiDASys software was used to process the data and find correlations between different parameters [53]. An uncertainty weighted least-squares linear regression (UWLR) for arsenic and other parameters was conducted (Figure 13, Figure 14 and Figure 15). According to Pearson (1896), correlation analysis is used to quantify and set up connections between two factors. The correlation coefficient of less than 0.5 represents low correlation, 0.5 signifies good correlation, and more than 0.5 denotes significant correlation. A solid link between two factors is displayed by a high correlation coefficient (close to +1 or −1), while a correlation coefficient of about zero represents that there is no relationship. Based upon the correlation coefficient “r”, the relationship between two parameters designed on an XY scatter diagram can be determined to be positive or negative [73]. 

It has been reported that arsenic and fluoride co-occur, but this does not necessarily imply a positive correlation between the two contaminants. The key influencers of the strength of the co-occurrence are seasonality, environment, and climatic conditions. Moreover, existing primary ion and dissolved organic matter also affect the release and enrichment of As-F in the aquifer system [74,75,76]. In this study, there is a correlation between these elements: in 2005, for arsenic and fluoride, mixed flow presented the highest correlation coefficient of all of the flows at 0.8004; on the other hand, the highest correlation coefficient in 2015 was for regional flow at 0.7135. Figure 13 presents the relationship between fluoride and arsenic in the present study. High salinity, along with the presence of high As and F concentrations in irrigation waters, constitutes a strong limitation for agricultural productivity that can lead to a decrease in sensitive crop yields [76]. 

There is a correlation between the water quality index and arsenic concentrations. The mixed flow presented the highest correlation coefficients of all of the flows for both 2005 and 2015 at 0.9880 and 0.9972, respectively. Figure 14 presents the relationship between the WQI and arsenic in the present study. 

There is a correlation between SAR and arsenic. In 2005, intermediate flow presented the highest correlation coefficient of all of the flows at 0.7182. On the other hand, the highest correlation coefficient in 2015 was observed for mixed flow at 0.9930. Figure 15 presents the relationship between these parameters in the present study. 

Because there is a correlation between the calculated parameters—the WQI and the SAR—this reinforces the findings and scientific contribution of this research in terms of the importance of monitoring and studying these polluting elements for the health of the population.

## 4. Conclusions

Groundwater is one of the most important reservoirs for supply of water with different uses in semi-arid and arid areas of the world, particularly in Mexico, where there is considerable research interest in its evolution and behavior. In this research, a hydrogeochemical analysis of groundwater was carried out to identify the present flow systems. Based on these, evolution of groundwater quality for human consumption and irrigation purposes was estimated. The results indicate that the predominant flow systems are intermediate and regional.

The hydrogeochemical facies revealed that nature of the water for the different flow systems considered is Na^+^ > Ca^2+^ > Mg^2+^ > K^+^. This is confirmed in the Piper trilinear diagram delineating the hydrogeochemical facies in this study region, and it was shown that most of the samples belong to the calcium, sodium, and/or magnesium bicarbonate types.

The WQI index used to characterize the quality of drinking water and its comparison with the WHO, US EPA, Mexican, and BIS standards for flow systems indicate that it complies with the parameters considered in them; however, for nitrate and arsenic, they do not comply with the limits established in the WHO for monitoring in 2005. In 2015, a deterioration in quality with respect to nitrates and arsenic was observed, thereby not complying with the WHO and USEPA standards for intermediate and regional flows. On the other hand, the mixed flow does not comply with the Mexican standard. This suggests that consuming water with these characteristics can have a harmful effect on human health. In general, according to the WQI index, the groundwater of this region is categorized as excellent an index <50. A significant contribution of research is the evaluation of water quality via the comparison of the WQI without arsenic and that with it, which indicates that this parameter deteriorates the index in 2005 and 2015, presenting in this last year samples in the classification of “unacceptable” with values of >300.

According to the water quality indices, most of the agricultural irrigation samples in the different flow systems in 2005 and 2015 were suitable for groundwater irrigation. On the basis of the CE indices considering C_2_S_1_, for water with restrictions for certain horticultural crops, the SAR was mostly between the values of 10 and 18, categorizing this water as “good"”; the EC only presented values >2250, categorizing the water as “unsuitable”. Samples with a percentage of Na between 40 and 80% were deemed “permissible”; almost all MH samples within the desirable classification had values less than 50%; and PI values >75 corresponded to the “suitable” category. All samples were suitable for irrigation according to the SAR and PI indices. However, deterioration in each of these parameters was observed for 2015, which demonstrates the importance of constantly monitoring water quality.

An uncertainty weighted least-squares linear regression (UWLR) model was used to conduct a new weighted linear regression procedure based on estimates of total uncertainty, and it is considered a good alternative, because the use of uncertainty has a probability connotation, a strict confidence level of 99%. A correlation between arsenic, fluoride, water quality index, and SAR was identified for all flow systems in both 2005 and 2015. The highest correlations in 2005 for were fluor in the mixed flow (0.8004), for WQI in mixed flow (0.9880), and for the SAR intermediate flow (0.7182). On the other hand, highest correlations in 2015 were for fluor in regional flow (0.7135), for WQI in mixed flow (0.9972), and for SAR the mixed flow (0.9930), which suggests that anthropogenic or natural origin of these should continue to be investigated. On this basis, better control of groundwater quality policies regarding the main source of water in this region can be developed, thereby mitigating the direct effects that deterioration in quality have on the health of the entire population and slowing down socionatural development.

Future research should focus on continuing to evaluate water quality indices that include more decision elements to avoid risks in the population that drinks this water or consumes food produced with it. Moreover, the identification of the origins of parameters such as arsenic, iron, and fluoride that are considered to determine water quality is a priority, as it will allow for the application of technologies to control these. Additionally, it will enable the development of new, environmentally friendly, and inexpensive technologies for the treatment of groundwater and reduction of the values of such parameters. All these investigations in order to prevent diseases caused by prolonged exposure to various water pollutants that can potentially also reach the food of the daily intake through irrigation in agriculture.

## Figures and Tables

**Table 1:** Comparison of parameter concentrations (mg/L).

Parameters	WHO Standard	US EPA	Mexican Official Norm NOM-127-SSA1-1994	Bureau of Indian Standards (BIS)
pH	8.5	6.5–8.4	6.5–8.5	6.5–8.6
TDS	500	500	500	2000
Cl^−^	250	250	250	1000
SO4^−2^	250	250	250	400
Total hardness	180	180	500	600
F^−^	1.5	4	1.5	1.5
As	0.01	0.01	0.025	0.05

**Table 2:** The weight of each chemical parameter considered in this study.

Parameter	*W_i_*
As	5
pH	3
TDS	5
Cl^−^	5
SO_4_^−2^	5
Na^+^	4
K^+^	2
HCO_3_^−^	1
Ca^2+^	3
Mg^2+^	3
F^−^	5

**Table 3:** Permissible limits for groundwater irrigation.

Permissible Limits for Agricultural Irrigation from Groundwater		
Index	Range	Classification
EC (µs/cm)	<250	Excellent
	250–750	Good
	750–2250	Doubtful
	>2250	Unsuitable
SAR (meq/L)	<10	Excellent
	10–18	Good
	18–26	Doubtful
	>26	Unsuitable
%Na	<20	Excellent
	20–40	Good
	40–60	Permissible
	60–80	Doubtful
	>80	Unsuitable
MH (%)	<50	Desirable
	>50	Undesirable
PI (%)	<25	Unsuitable
	25–75	Moderately suitable
	>75	Suitable

**Table 4:** Clustering of flows as a result of cluster analysis for the year 2005 (TDS = total dissolved solids, concentration mg/L; modified from Avila et al., 2018 [43]).

Flow	Statistics Data	T°C	pH	TDS	HCO_3_^−^	Cl^−^	SO_4_^2−^	F^−^	Na^+^	K^+^	Ca^2+^	Mg^2+^	As (µg/L)
Regional	Min	20.00	6.36	152.00	121.51	5.42	10.00	0.00	23.8	2.70	10.30	0.30	2.49
	Max	29.00	8.55	1260.00	1439.21	223.38	1080.00	0.10	612.50	26.00	237.00	108.00	43.70
	Mean	25.34	7.75	271.90	285.47	20.40	94.20	0.84	79.43	10.38	52.96	14.18	17.85
	Variance	4.82	0.30	73,168.85	50,657.01	1536.48	38,658.80	0.35	12,831.81	23.30	1458.32	475.41	101.45
	Desv	2.19	0.54	197.49	225.84	39.20	196.62	0.54	113.27	3.58	38.18	21.80	10.07
	N	32.00	32.00	32.00	32.00	32.00	32.00	32.00	32.00	32.00	32.00	32.00	32.00
Intermediate	Min	16.20	6.09	168.00	72.90	0.92	12.00	0.03	9.00	2.70	15.50	1.00	4.40
	Max	30.00	8.40	427.00	473.16	81.23	167.50	1.82	145.00	27.70	92.60	54.00	61.40
	Mean	25.02	7.84	64.63	74.85	12.07	45.77	0.42	45.94	10.50	50.53	13.74	15.36
	Variance	4.69	0.19	3592.14	3348.85	127.75	804.73	0.20	683.30	20.67	157.20	139.42	109.25
	Desv	2.14	0.44	249.15	257.55	11.30	27.95	0.62	57.46	26.35	4.60	13.22	10.33
	N	44.00	44.00	44.00	44.00	44.00	44.00	44.00	44.00	44.00	44.00	44.00	44.00
Local	Min	17.40	7.80	61.00	36.45	3.51	8.00	0.00	8.20	4.70	9.60	1.10	0.49
	Max	19.70	8.28	110.00	374.59	10.60	28	0.00	25.00	8.20	50.30	32.60	3.29
	Mean	18.30	8.11	144.66	199.40	6.97	21	0.00	18.40	5.90	29.23	15.10	1.93
	Variance	1.46	0.07	14,562.33	14,398.54	12.58	127.00	0.00	80.28	3.97	415.63	253.26	1.96
	Desv	1.20	0.26	120.67	119.90	3.54	11.26	0.00	8.95	1.99	20.38	15.91	1.40
	N	3.00	3.00	3.00	3.00	3.00	3.00	3.00	3.00	3.00	3.00	3.00	5.00
Mixture	Min	21.10	6.48	139.00	197.15	4.06	9.00	0.10	14.60	5.70	28.20	2.50	4.70
	Max	29.20	8.13	391.00	473.16	18.28	110.00	1.86	98.00	13.20	117.60	25.20	44.50
	Mean	23.81	7.18	241.15	251.08	10.33	35.65	1.03	44.25	9.99	46.06	10.61	18.57
	Variance	3.81	0.23	3092.45	3385.65	10.87	503.61	0.17	300.59	4.67	326.98	50.75	69.13
	Desv	1.95	0.47	55.60	58.18	3.30	22.44	0.35	17.33	2.16	18.08	7.12	8.31
	N	20.00	20.00	20.00	20.00	20.00	20.00	20.00	20.00	20.00	20.00	20.00	20.00

**Table 5:** Clustering of flows as a result of cluster analysis for the year 2015 (TDS = total dissolved solids, concentration mg/L; modified from Avila et al., 2018).

Flow	Statistics Data	T°C	pH	TDS	HCO_3_^−^	Cl^−^	SO_4_^2−^	F^−^	Na^+^	K^+^	Ca^2+^	Mg^+2^	As (µg/L)	Depth (m)
Regional	Min	22.50	6.71	137.20	134.69	8.44	2.00	0.44	17.64	1.06	1.85	0.10	3.64	10.00
	Max	37.00	8.89	347.90	363.07	35.73	82.00	5.40	106.73	17.62	63.97	0.095	1219.00	274.00
	Mean	27.68	7.39	319.18	227.25	50.68	82.12	1.33	61.34	11.03	4905	29.27	20.51	159.57
	Variance	10.44	0.29	93,344.49	2708.69	28.41	289.58	0.85	509.76	28.08	122.99	64.78	382.10	3616.61
	Desv	3.22	0.46	48.06	52.55	5.41	17.02	0.92	22.82	3.47	11.02	7.99	19.55	60.14
	N	47.00	47.00	47.00	47.00	47.00	47.00	47.00	47.00	47.00	47.00	47.00	47.00	47.00
Intermediate	Min	19.10	6.43	58.80	133.71	8.44	6.00	0.39	12.41	2.03	5.78	1.15	6.25	80.00
	Max	40.10	8.19	721.77	816.18	268.00	230.00	4.25	181.30	23.18	198.92	50.18	241.30	300.00
	Mean	26.04	7.32	253.46	227.08	36.39	35.10	1.04	47.58	10.20	40.93	16.26	21.70	177.49
	Variance	11.22	0.27	8927.19	3415.82	1308.79	1228.18	0.38	700.10	17.57	281.29	129.29	468.89	2859.45
	Desv	3.36	0.49	121.09	104.52	55.42	35.05	0.61	35.18	4.61	29.48	11.97	20.99	53.47
	N	49.00	49.00	49.00	49.00	49.00	49.00	49.00	49.00	49.00	49.00	49.00	49.00	49.00
Mixture	Min	21.70	6.59	98.00	132.74	8.93	2.00	0.39	18.13	3.99	9.41	0.83	5.19	87.00
	Max	30.20	8.34	392.00	420.90	31.76	72.00	3.60	166.13	13.38	47.24	40.76	120.65	210.00
	Mean	25.99	7.68	217.43	213.76	14.91	26.35	1.02	39.58	8.93	29.23	13.39	25.30	168.79
	Variance	5.39	0.18	5076.45	4559.01	37.10	337.82	0.08	8.25	456.87	114.11	136.97	2615.75	1950.18
	Desv	2.21	0.38	72.02	66.74	5.66	16.97	0.67	33.12	2.95	9.77	11.12	51.21	45.00
	N	20.00	20.00	20.00	20.00	20.00	20.00	20.00	20.00	20.00	20.00	20.00	20.00	20.00

**Table 6:** Comparison of the average values of different parameters and each flow system with the permissible limits with respect to different regulations.

**2005**																				
**Parameters**					**WHO standard**				**US EPA**				**Mexican Official norm NOM-127-SSA1-1994**				**Bureau of Indian Standards (BIS)**			
**Mean values**																				
	Reg.	Int.	Loc.	Mix.	Reg.	Int.	Loc.	Mix.	Reg.	Int.	Loc.	Mix.	Reg.	Int.	Loc.	Mix.	Reg.	Int.	Loc.	Mix.
pH	7.75	7.84	8.11	7.18	✔	✔	✔	✔	✔	✔	✔	✔	✔	✔	✔	✔	✔	✔	✔	✔
TDS	316.90	246.76	144.66	241.15	✔	✔	✔	✔	✔	✔	✔	✔	✔	✔	✔	✔	✔	✔	✔	✔
Cl^−^	20.40	12.07	6.97	10.33	✔	✔	✔	✔	✔	✔	✔	✔	✔	✔	✔	✔	✔	✔	✔	✔
SO_4_^2−^	94.20	45.77	21.00	35.65	✔	✔	✔	✔	✔	✔	✔	✔	✔	✔	✔	✔	✔	✔	✔	✔
NO_3−_	0.70	0.63	0.74	0.76	X	X	X	X	✔	✔	✔	✔	✔	✔	✔	✔	✔	✔	✔	✔
F	0.85	0.61	0.00	0.98	✔	✔	✔	✔	✔	✔	✔	✔	✔	✔	✔	✔	✔	✔	✔	✔
As	0.01	0.01	0.00	0.02	X	X	✔	X	✔	✔	✔	✔	✔	✔	✔	✔	✔	✔	✔	✔
**2015**																				
**Parameters**					**WHO standard**				**US EPA**				**Mexican Official norm NOM-127-SSA1-1994**				**Bureau of Indian Standards (BIS)**			
**Mean values**																				
	Reg.	Int.	Loc.	Mix.	Reg.	Int.	Loc.	Mix.	Reg.	Int.	Loc.	Mix.	Reg.	Int.	Loc.	Mix.	Reg.	Int.	Loc.	Mix.
pH	7.39	7.38	-	7.72	✔	✔	-	✔	✔	✔	-	✔	✔	✔	-	✔	✔	✔	-	✔
TDS	319.18	235.64	-	223.85	✔	✔	-	✔	✔	✔	-	✔	✔	✔	-	✔	✔	✔	-	✔
Cl^−^	50.68	27.08	-	15.34	✔	✔	-	✔	✔	✔	-	✔	✔	✔	-	✔	✔	✔	-	✔
SO_4_^2−^	82.12	35.08	-	26.35	✔	✔	-	✔	✔	✔	-	✔	✔	✔	-	✔	✔	✔	-	✔
NO_3−_	2.92	2.90	-	2.93	X	X	-	X	X	X	-	X	✔	✔	-	✔	✔	✔	-	✔
F	1.33	1.02	-	0.83	✔	✔	-	✔	✔	✔	-	✔	✔	✔	-	✔	✔	✔	-	✔
As	0.02	0.02	-	0.03	X	X	-	X	X	X	-	X	✔	✔	-	X	✔	✔	-	✔

✔ within limits; X limits exceeded.

## Data Availability

The data presented in this study are available in the article.
